# Correction: Kay et al. Effects of Water and Wind Stress on Phytochemical Diversity, Cannabinoid Composition, and Arthropod Diversity in Hemp. *Plants* 2025, *14*, 474

**DOI:** 10.3390/plants15060956

**Published:** 2026-03-20

**Authors:** Ericka R. Kay, Casey S. Philbin, Lora A. Richards, Matthew L. Forister, Christopher Jeffrey, Lee A. Dyer

**Affiliations:** Department of Biology, University of Nevada Reno, 1664 N Virginia St., Reno, NV 89557, USA; ekay@unr.edu (E.R.K.); cphilbin@unr.edu (C.S.P.); lorar@unr.edu (L.A.R.); mforister@unr.edu (M.L.F.); cjeffrey@unr.edu (C.J.)

## Figure/Table Legend

In the original publication [1], there was a mistake in the legend for Figures 2 and 4 as follows. Figure 2 caption: “Stress effects (water deficit and wind stress) increased diversity across both varieties.” Figure 4 caption “…direct negative effects of flooding and wind stress on plant height and CBD levels.” The correct sentences within these legends are as follows. Figure 2: Stress effects (water deficit and wind stress) decreased diversity across both varieties. Figure 4: …direct negative effects of flooding and wind stress on plant height.

## Text Correction

There was an error in the original publication on Section 2.1, 4th sentence: “Stress responses caused by water deficit and wind damage were weaker than variety, but both forms of stress caused higher levels of phytochemical diversity (Figure 3)”. Section 2.3, 6th & 7th sentence: “CBD had a positive direct effect on arthropod richness (0.09), suggesting that higher CBD levels may promote greater arthropod diversity. Direct negative effects of flood (−0.16) and wind stress (−0.71) on plant height indirectly affected CBD levels and arthropod richness.” A correction has been made to each of these sections as follows. Section 2.1: Stress responses caused by water deficit and wind damage were weaker than those of the variety, but both forms of stress caused lower levels of phytochemical diversity (Figure 3). Section 2.3: CBD had a negative direct effect on arthropod richness (−0.2), suggesting that higher CBD levels may promote greater arthropod diversity. Direct negative effects of flood (−0.16) and wind stress (−0.71) on plant height indirectly affected arthropod richness.

The authors state that the scientific conclusions are unaffected. This correction was approved by the Academic Editor. The original publication has also been updated.
