# Supplementary material for: Effects of Water and Wind Stress on Phytochemical Diversity, Cannabinoid Composition, and Arthropod Diversity in Hemp
Source: Plants (Basel). 2025 Feb 5;14(3):474. doi: 10.3390/plants14030474 (PMC11819868; doi:10.3390/plants14030474)
Supplement: Supplementary file 1 [file plants-14-00474-s001.zip › plants-3381958-supplementary.pdf]

Effects of water and wind stress on phytochemical diversity, cannabinoid composition, and arthropod diversity in hemp

Ericka R. Kay, Casey S. Philbin, Lora A. Richards, Matthew L. Forister, Christopher Jeffrey, Lee A. Dyer

University of Nevada Reno; 1664 N Virginia St; Reno, NV 89557

SUPPLEMENTS

Section S1: experimental design

Section S2: detailed cannabinoid results

Section S3: additional arthropod results

Section S4: complete R scripts, including alternative models

Section S1: Experimental design

Table S1. Summary of experimental design (*n* = 150 plants)

2020												
Variety	Cherry Wine (108 plants)						Lifter (42 plants)					
Wind stress	No (86)			Yes (22)			No (24)			Yes (18)		
Water treatment	0.5	1	2	0.5	1	2	0.5	1	2	0.5	1	2
Number of plants	28	27	31	8	8	6	7	9	8	7	6	5

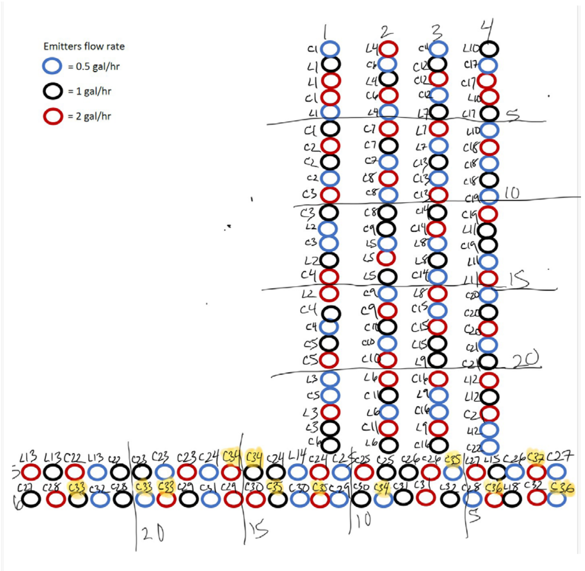

Figure S1. Summary of experimental design (*n* = 150 plants)

Section S2: Detailed cannabinoid results

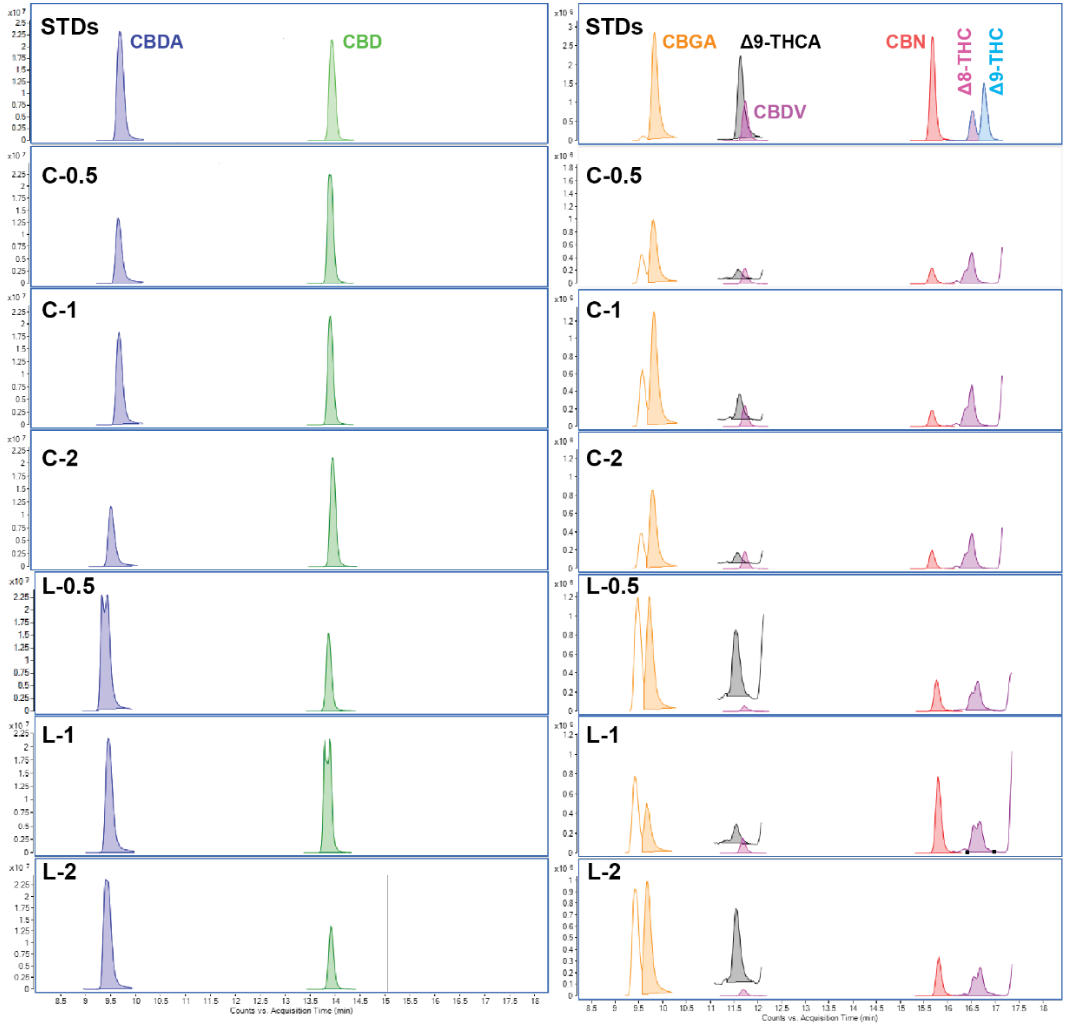

Figure S2. EICs of CBDA and CBD (left) and CBGA, Δ9-THCA, CBDV, CBN, Δ8-THC, and Δ9-THC (right) standards and characteristic sample chromatograms for each water treatment (0.5, 1, 2 gph) within each variety (C, Cherry Wine; L, Lifter). The EIC for Δ9-THC was shown to demonstrate we were able to resolve Δ8- and Δ9-THC using our method, but we did not observe Δ9-THC in any of our samples, so it was not quantified.

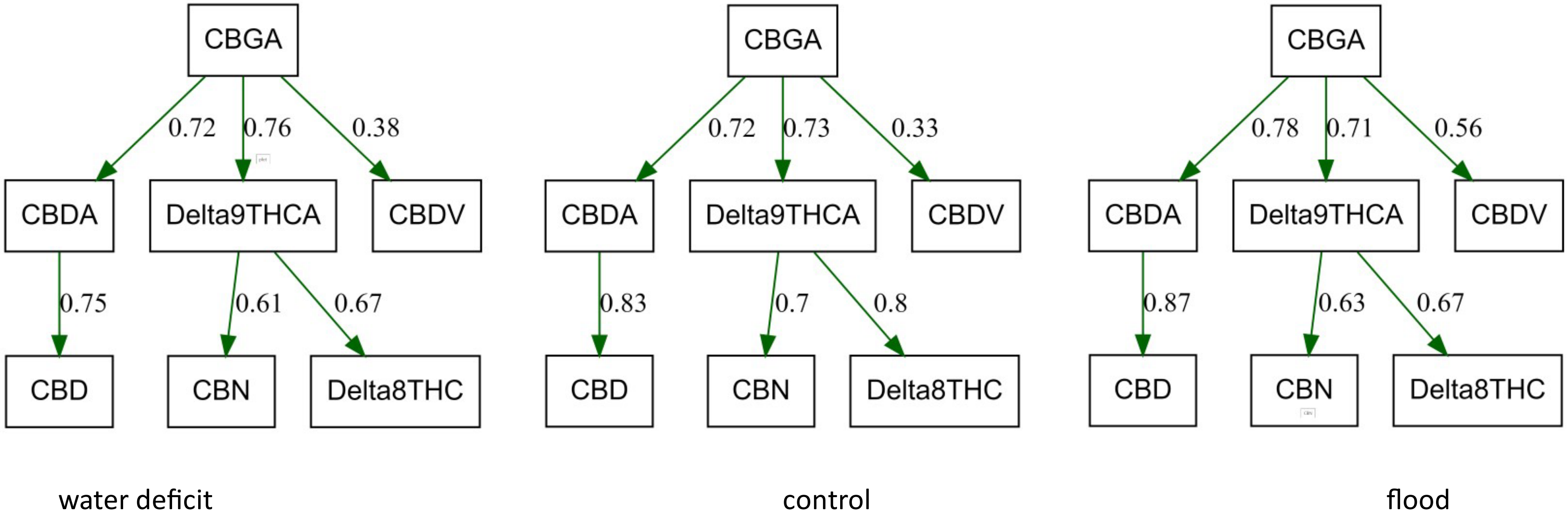

Figure S3. Best fit structural equation models (SEM) depicting putative causal relationships among focal cannabinoids. SEM allowed us to evaluate and compare the relative importance of different causal pathways (represented as arrows in the models) across conditions. These models were informed by prior knowledge of cannabinoid biosynthesis (Figure 1) and were designed to evaluate how water treatments influence cannabinoid concentrations. The SEMs support hypotheses of shifts in causal pathways for cannabinoids in response to the different levels of water treatment.

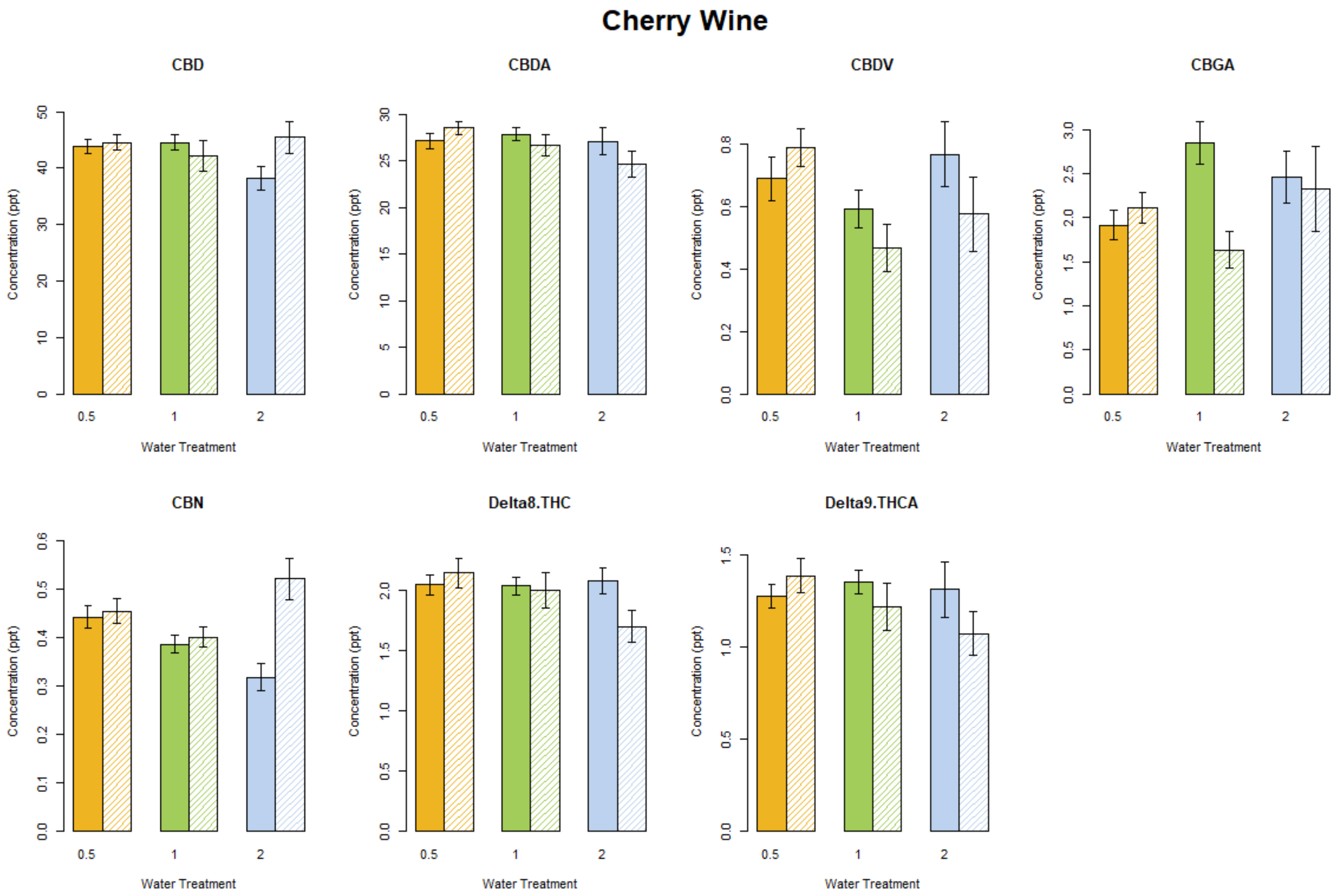

Cherry Wine
CBD
CBDA
CBDV
CBGA
CBN
Delta8.THC
Delta9.THCA
Concentration (ppt)
Water Treatment
0.5
1
2

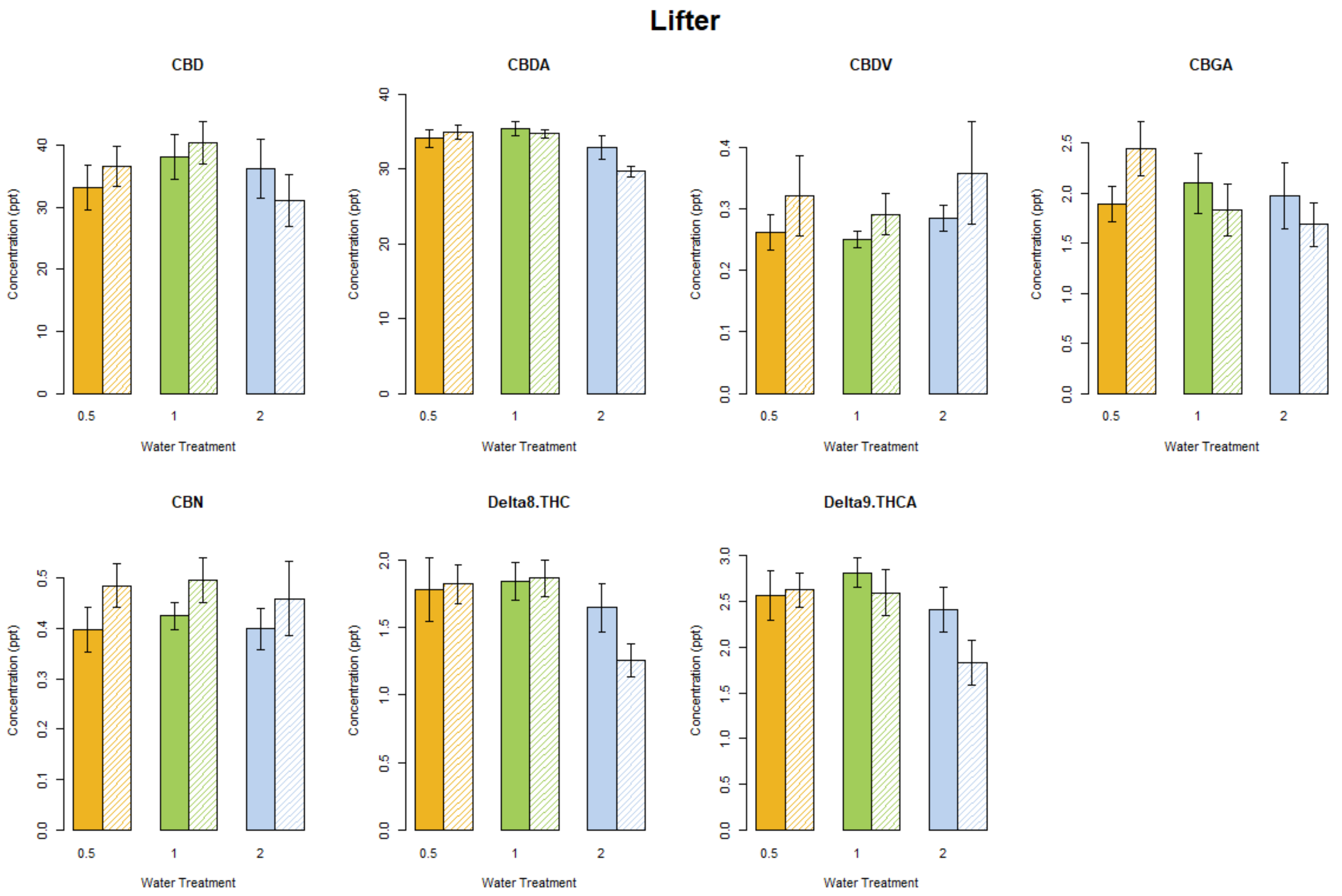

Figure S4. Graphical summary displaying central tendencies (means) and dispersions (±1 standard error of the mean) of cannabinoid concentrations (μM/g) across water treatments and wind stress for Cherry Wine (upper figure) and Lifter (lower figure). Hashed bar indicated wind stressed plants. The means and standard errors are averaged across variety and flower location. For THCA compounds, only the Δ9-THCA standard was available; therefore, putative Δ8-THC and Δ9-THCA concentrations could only be semi-quantified based on Δ9-THCA calibration curve.

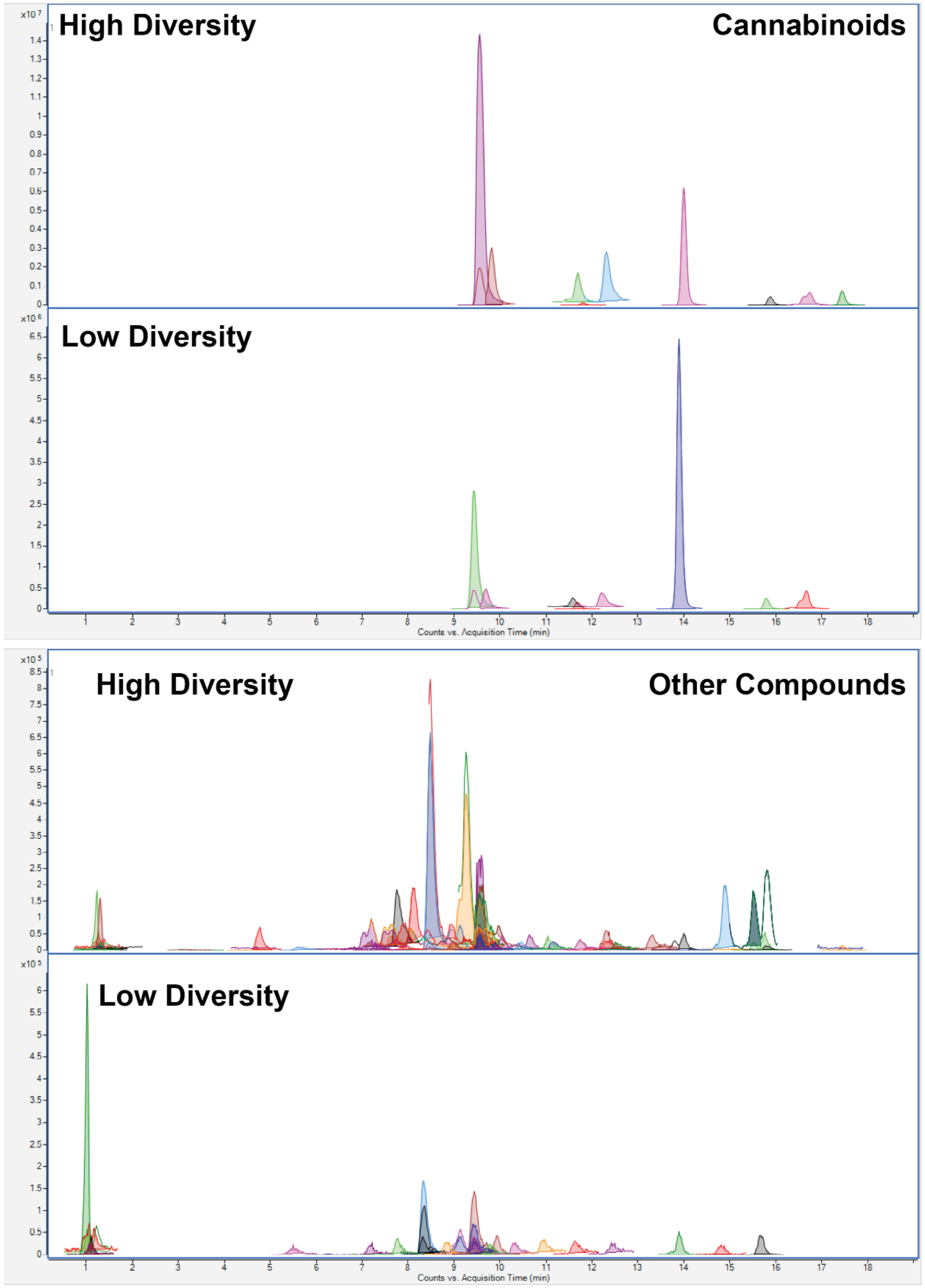

Figure S5. Extracted ion chromatograms of high and low chemical diversity samples. Samples were among the highest and lowest diversity measures for both Simpson and Shannon diversity.. Cannabinoids are presented separately from all other phytochemicals due do the large differences in scale.

Table S2. Cannabinoid LC-MS properties and calibration curve parameters averaged over daily (n = 16) calibration curves.

Cannabinoid Properties					Calibration Curve Parameters							
					Response†		Linearity (R^2)		LOQ (ppt)*		LOD (ppt)*	
Name	Formula	[M-H]-	ppm diff	RT	mean	s.d.	mean	s.d.	mean	s.d.	mean	s.d.
CBDV	C19H26O2	285.1863	0.93	11.79	11.1	0.17	0.996	0.001	0.148	0.035	0.049	0.011
Δ8-THC	C21H30O2	313.2176	0.78	16.77	15.3	0.35	0.999	0.001	0.069	0.016	0.023	0.005
CBD	C21H30O2	313.2177	1.16	14.02	8.79	0.14	0.996	0.001	0.234	0.037	0.078	0.012
CBN	C21H26O2	309.1866	1.71	15.90	4.02	0.12	0.996	0.001	0.465	0.05	0.155	0.018
CBDA	C22H30O4	357.2077	1.26	9.55	3.46	0.08	0.996	0.001	0.567	0.126	0.189	0.04
CBGA	C22H32O4	359.2232	0.64	9.77	3.6	0.09	0.996	0.001	0.592	0.137	0.197	0.05
Δ9-THCA	C22H30O4	357.2073	-0.53	11.64	4.11	0.12	0.998	0.001	0.409	0.057	0.136	0.019

* based on 10.0 mg extraction

† μM/umbelliferone equivalents

Table S3. Cannabinoid concentrations (ppt) summaries, including mean and standard deviation, by experimental factor. For THCA compounds, only the Δ9-THCA standard was available; therefore, putative Δ8-THC and Δ9-THCA concentrations could only be semi-quantified based on Δ9-THCA calibration curve.

Experimental Factors			CBD		CBDA		CBDV		CBGA		CBN		Δ8 THC		Δ9 THCA	
Flower	Var	Water	mean	s.d.	mean	s.d.	mean	s.d.	mean	s.d.	mean*	s.d.	mean	s.d.	mean	s.d.
lower	C	0.5	44.6	9.98	26.6	5.70	0.452	0.429	1.30	0.888	d	-	1.98	0.668	1.16	0.484
lower	C	1	43.6	10.6	26.8	5.88	0.546	0.422	1.60	1.12	d	-	2.01	0.587	1.18	0.461
lower	C	2	45.0	9.99	26.6	5.93	0.379	0.382	2.23	1.43	d	-	1.95	0.563	1.24	0.470
lower	L	0.5	39.0	14.0	35.1	3.37	0.219	0.163	1.60	0.749	0.471	0.201	1.83	0.777	2.57	0.950
lower	L	1	39.7	13.5	34.3	6.00	0.287	0.299	1.83	1.10	0.472	0.175	1.81	0.664	2.48	0.940
lower	L	2	35.8	14.7	33.5	4.87	0.219	0.217	1.88	1.12	d	-	1.63	0.647	2.43	0.914
upper	C	0.5	41.3	11.4	27.5	6.01	0.500	0.490	2.33	1.19	d	-	2.03	0.650	1.32	0.526
upper	C	1	42.5	8.87	29.6	4.23	0.652	0.511	2.72	1.16	d	-	2.25	0.939	1.56	0.750
upper	C	2	44.4	10.7	28.0	5.04	0.416	0.451	3.24	2.10	d	-	2.02	0.608	1.37	0.493
upper	L	0.5	34.9	12.3	33.7	3.10	0.234	0.127	2.13	0.817	d	-	1.82	0.577	2.59	0.969
upper	L	1	33.3	14.9	34.0	2.59	0.187	0.129	2.68	1.08	d	-	1.69	0.567	2.59	0.688
upper	L	2	35.0	14.3	33.0	3.76	0.205	0.14	2.00	0.981	d	-	1.61	0.508	2.44	0.790

*d indicates cannabinoid was above LOD but below LOQ.

Section S3: additional arthropod results

Fig S6a. Cherry wine

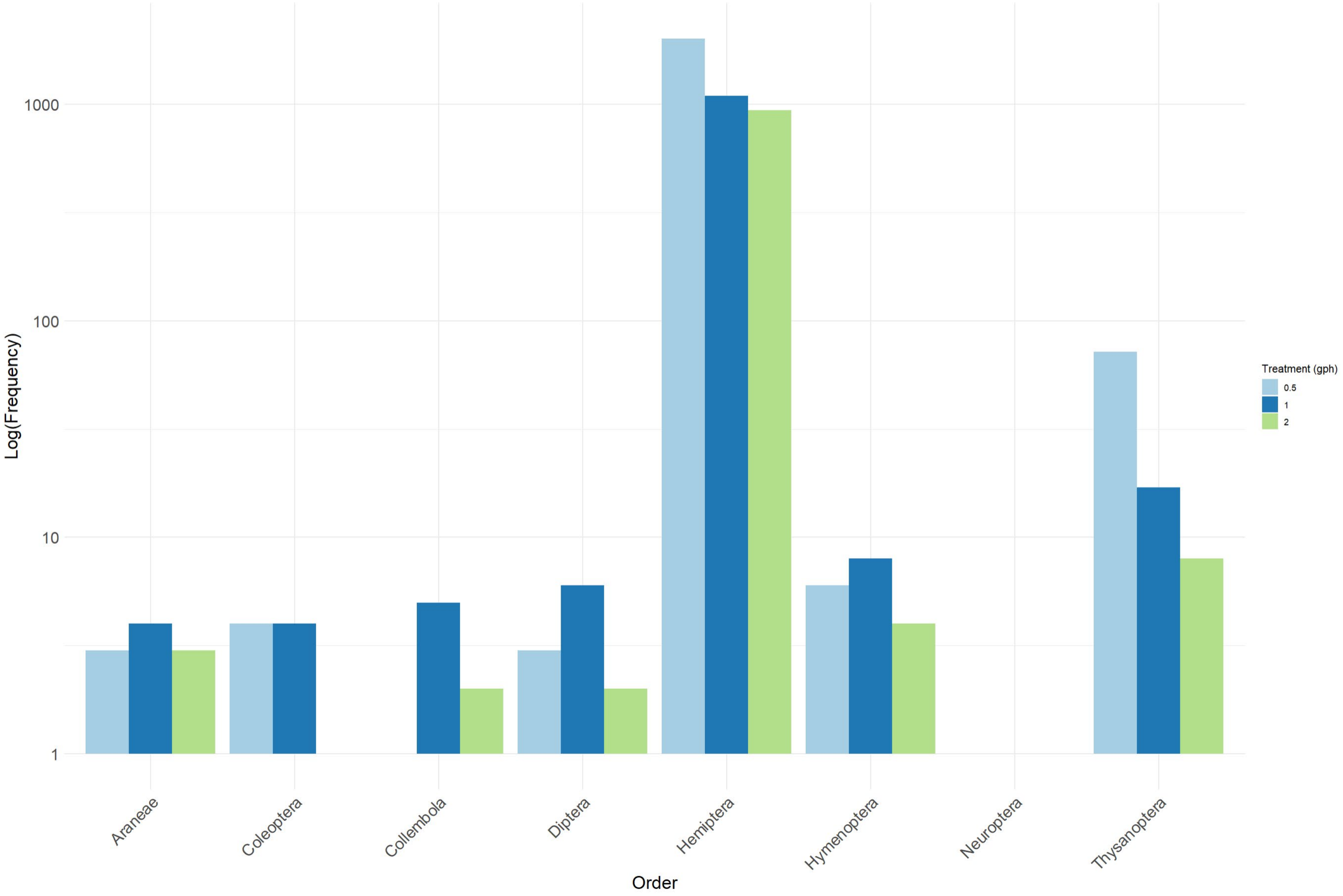

Fig S6a Lifter

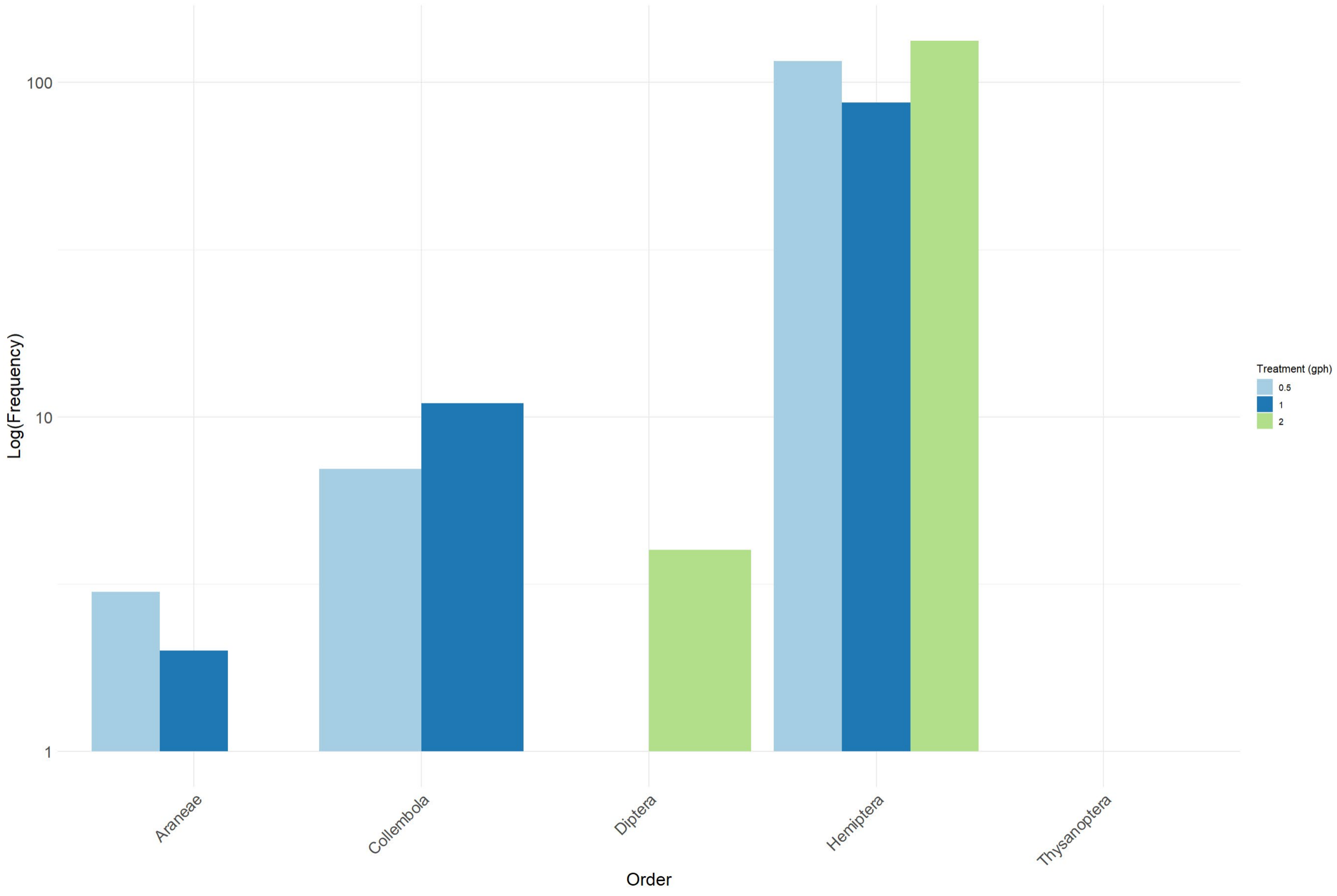

Fig S6b Cherry Wine

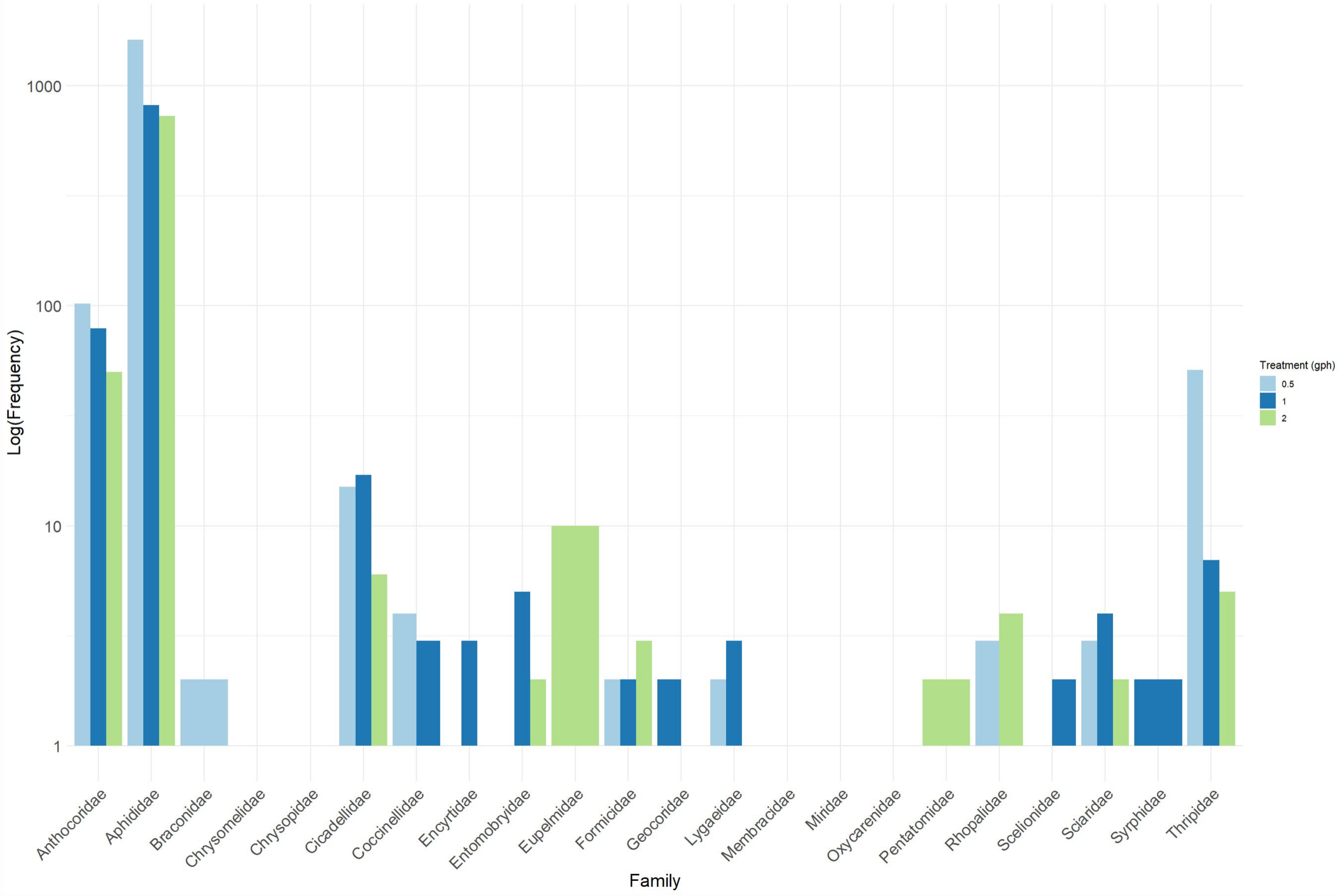

Fig S6b Lifter

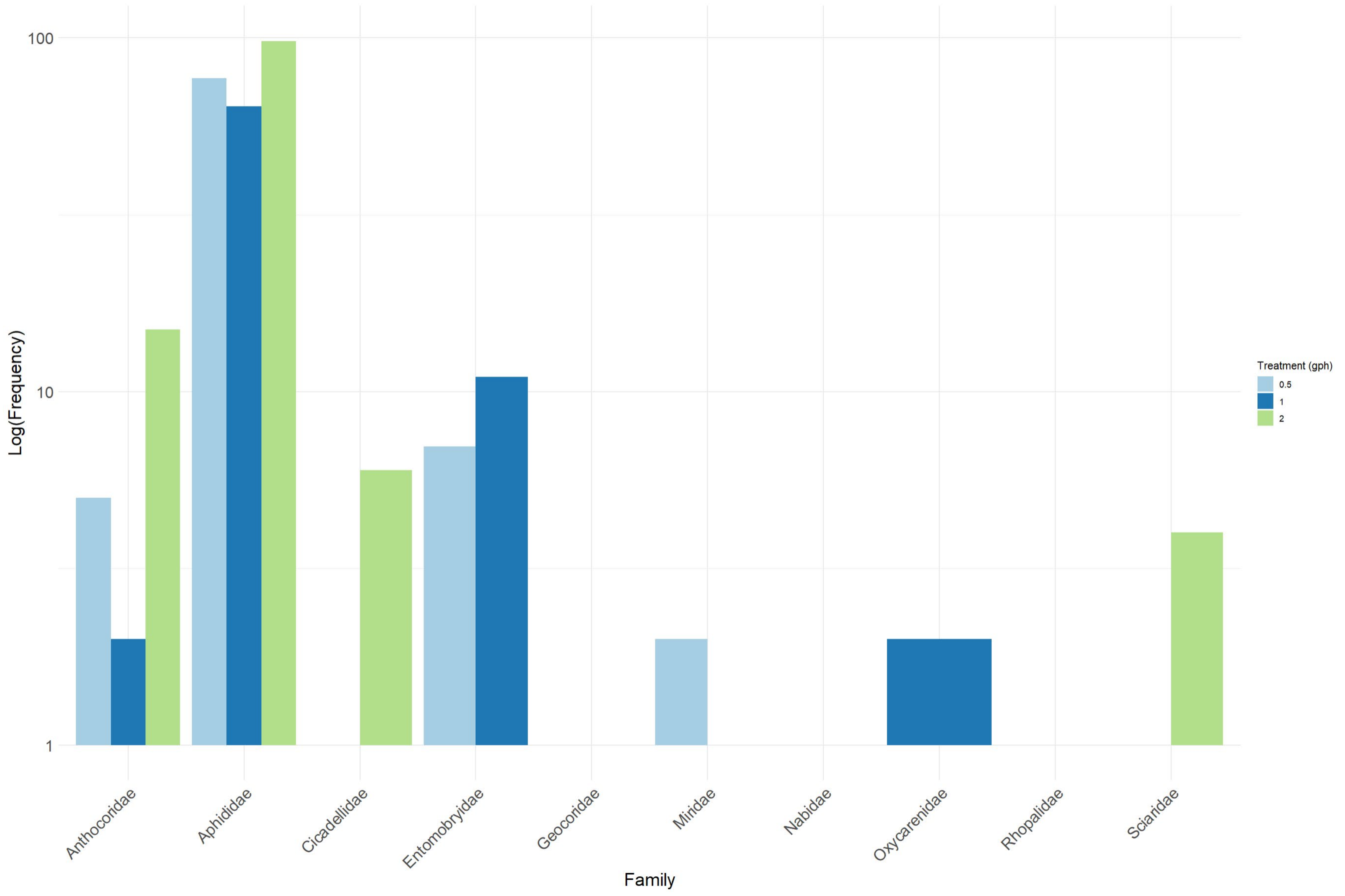

Figure S6. Log frequency distributions of arthropod orders (S2a) and families (S2b) collected from the flowers of experimental plants in 2020, categorized by treatment and variety. The upper bar charts of each figure represent Cherry Wine (C) clones, while the lower bar charts represent Lifter (L) clones. Taxa for which only one individual was found are included (and have no apparent bars). Diverse arthropod communities were observed, with the most abundant orders being Thysanoptera and Hemiptera. Herbivorous families dominated the collections, including Thripidae, Aphididae, and Cicadellidae, with additional contributions from Chrysomelidae (Coleoptera) and Rhopalidae (Hemiptera). Predaceous and parasitoid families, such as Coccinellidae (Coleoptera), Anthocoridae and Pentatomidae (Hemiptera), and Braconidae and Encyrtidae (Hymenoptera), were also well-represented. Omnivorous taxa included Formicidae (Hymenoptera) and Syrphidae (Diptera), the latter of which are predaceous during their larval stage. The three levels of water treatment effects are visualized as with light blue, dark blue, and green representing 0.5, 1, and 2 gallons per hour respectively.

Section S4: Complete R scripts with alternative models

```
#########################
##Analyses for Ericka Kay's drought MS number one
#########################

setwd("D:/Rprojects/Cannabis")
library(lavaan)
library(lavaanPlot)
library(dplyr)
library(stringr)
library(ggplot2)
library(vegan)
library(psych)
library(ggcorrplot)
library(lme4)


##FUNCTION for HILL NUMBERS #################
calculate_hill_numbers_row <- function(row) {
  # Keep only numeric values for the row
  row <- as.numeric(row)

  if (sum(row) == 0) {
    return(c(D0 = NA, D1 = NA, D2 = NA))  # Skip rows with all zeros
  }

  # Hill numbers calculation
  D0 <- sum(row > 0)  # Richness (Hill number 0)
  p <- row / sum(row)  # Proportions
  shannon <- -sum(p * log(p + 1e-10))  # Shannon (Hill number 1)
  D1 <- exp(shannon)
  D2 <- 1 / sum(p^2)  # Simpson (Hill number 2)

  return(c(D0 = D0, D1 = D1, D2 = D2))
}

#######CANNABINOIDS
data <- read.csv("EKHempDrought_CannabQuant_ISD_Calib_DWT.csv")
data[is.na(data)] <- 0
```

```
data[, -1] <- scale(data[, -1])
if (!"identifier" %in% colnames(data)) {
  data <- data %>% mutate(identifier = X)
}
data <- data %>%
  mutate(
    treatment = str_extract(identifier, "(?<=trt_)(\\d+\\.\\d+|\\d+)") %>%
      str_replace("^05$", "0.5") %>%
      as.numeric(),
    variety = str_extract(identifier, "(?<=var_)\\w+"),
    replicate = as.numeric(str_extract(identifier, "(?<=rep_)\\d+")),
    year = as.numeric(str_extract(identifier, "(?<=Drought\\.)\\d+")
  )
)
data_2019 <- data %>% filter(year == 2019)
data_2020 <- data %>% filter(year == 2020)

########SO NOW I AM JUST DOING ANALYSES FOR 2020. SEE OTHER SCRIPTS FOR BOTH YEARS INCLUDED
data <- data_2020

#PATH ANALYSIS!
model <- '
  CBGA  ~ 1  # CBGA is the precursor
  Delta9.THCA  ~ CBGA
  CBDA  ~ CBGA
  CBDV  ~ CBGA
  CBD  ~ CBDA
  CBN  ~ Delta9.THCA
  Delta8.THC ~ Delta9.THCA
'
fit <- sem(model, data = data)
summary(fit, standardized = TRUE)
lavaanPlot(model = fit,
           node_options = list(shape = "box", fontname = "Helvetica"),
           edge_options = list(color = "darkgreen"),
           coefs = TRUE,
           covs = FALSE,
           stand = TRUE)

###########SEM
```

```
# Split the data by the three treatment levels (0.5, 1, 2)
data05 <- data %>% filter(treatment == 0.5)
data1 <- data %>% filter(treatment == 1)
data2 <- data %>% filter(treatment == 2)
#################DROUGHT treatments
# Fit the model for each DROUGHT treatment level
fit05 <- sem(model, data = data05)
fit1 <- sem(model, data = data1)
fit2 <- sem(model, data = data2)
summary(fit05, standardized = TRUE)
summary(fit1, standardized = TRUE)
summary(fit2, standardized = TRUE)

lavaanPlot(model = fit05,
        node_options = list(shape = "box", fontname = "Helvetica"),
        edge_options = list(color = "darkgreen"),
        coefs = TRUE,
        covs = FALSE,
        stand = TRUE)

lavaanPlot(model = fit1,
        node_options = list(shape = "box", fontname = "Helvetica"),
        edge_options = list(color = "darkgreen"),
        coefs = TRUE,
        covs = FALSE,
        stand = TRUE)

lavaanPlot(model = fit2,
        node_options = list(shape = "box", fontname = "Helvetica"),
        edge_options = list(color = "darkgreen"),
        coefs = TRUE,
        covs = FALSE,
        stand = TRUE)

####VARIETY
data_L <- data_2020 %>% filter(variety == "L")
data_C <- data_2020 %>% filter(variety == "C")
fit_L <- sem(model, data = data_L)
fit_C <- sem(model, data = data_C)
summary(fit_L, standardized = TRUE)
```

```
summary(fit_C, standardized = TRUE)
lavaanPlot(model = fit_L,
        node_options = list(shape = "box", fontname = "Helvetica"),
        edge_options = list(color = "darkgreen"),
        coefs = TRUE,
        covs = FALSE,
        stand = TRUE)
lavaanPlot(model = fit_C,
        node_options = list(shape = "box", fontname = "Helvetica"),
        edge_options = list(color = "darkgreen"),
        coefs = TRUE,
        covs = FALSE,
        stand = TRUE)


##########################################################################################################
##BUGS - overall abundance, order richness, family hill numbers
bugs <- read.csv("UNR_bugs.csv")
colnames(bugs)[colnames(bugs) == "water_treatment"] <- "treatment"
bugs$year <- as.numeric(bugs$year)
bugs$treatment <- as.numeric(bugs$treatment)
bugs$replicate <- as.numeric(bugs$replicate)
bugs <- bugs[bugs$year != 2018, ]
bugs <- bugs[!is.na(bugs$order) & bugs$order != "", ]
abundance <- bugs %>%
  group_by(year, treatment, variety, replicate) %>%
  summarise(abundance = n())
richness <- bugs %>%
  group_by(year, treatment, variety, replicate) %>%
  summarise(richness = n_distinct(order))
bugs_family <- bugs[bugs$family != "", ]

######Hill numbers based on FAMILY of arthropod
filtered_bugs <- bugs_family %>%
    filter(!is.na(family) & family != "")
hill_numbers <- filtered_bugs %>%
    group_by(year, treatment, variety, replicate) %>%
    summarise(
        # Calculate D0: Number of distinct families (richness)
        D0 = n_distinct(family),
```

```
    # Calculate famabun: total abundance (number of specimens)
    famabun = n(),

    # Calculate Hill numbers
    p = famabun / sum(famabun),  # Proportions for Hill numbers
    D1 = exp(-sum(p * log(p + 1e-10))),  # Hill number 1 (Shannon diversity)
    D2 = 1 / sum(p^2),  # Hill number 2 (Simpson diversity)

    .groups = 'drop'  # Ungroup after summarise
  )

merged_data1 <- data %>%
  left_join(abundance, by = c("year", "treatment", "variety", "replicate")) %>%
  left_join(richness, by = c("year", "treatment", "variety", "replicate")) %>%
  left_join(hill_numbers, by = c("year", "treatment", "variety", "replicate"))

# I relaced NA with 0 and Inf with 10 for diversity columns
merged_data2 <- merged_data1 %>%
  mutate(
    abundance = replace(abundance, is.na(abundance), 0),
    richness = replace(richness, is.na(richness), 0),
    D0 = replace(D0, is.na(D0), 0),
    D1 = replace(D1, is.na(D1), 0),
    D2 = replace(D2, is.na(D2), 0),
    D1 = replace(D1, is.infinite(D1), 10),
    D2 = replace(D2, is.infinite(D2), 30)
  )

ChemDiv <- merged_data2
freq_table <- with(ChemDiv, table(year, variety, treatment))
print(freq_table)


##############################################ARTHROPOD SUMMARY
bugs_2020 <- bugs %>% filter(year == 2020)
bugs_2020 <- bugs_2020 %>%
  mutate(
    order = case_when(
      order == "Hemiptera?" ~ "Hemiptera",
```

```
    order == "Entomobryomorpha" ~ "Collembola",
    TRUE ~ order
  ),
  family = case_when(
    family == "Aphididae?" ~ "Aphididae",
    family == "Nysius" ~ "Lygaeidae",
    TRUE ~ family
  )
 )
bugs_order <- bugs_2020 %>% filter(!is.na(order))
bugs_family <- bugs_2020 %>% filter(!is.na(family))
bugs_order_summary <- bugs_order %>%
 group_by(treatment, variety, order) %>%
 summarize(frequency = n(), .groups = "drop")
bugs_family <- bugs_family %>%
 mutate(family = ifelse(family == "Coccinelidae", "Coccinellidae", family))
bugs_family <- bugs_family %>%
 mutate(family = ifelse(is.na(family) | family == "", "Missing", family))
bugs_order_var_C <- bugs_order %>% filter(variety == "C")
bugs_order_var_L <- bugs_order %>% filter(variety == "L")
bugs_family_var_C <- bugs_family %>% filter(variety == "C" & family != "Missing")
bugs_family_var_L <- bugs_family %>% filter(variety == "L" & family != "Missing")
treatment_colors <- c("0.5" = "#A6CEE3", "1" = "#1F78B4", "2" = "#B2DF8A")
axis_text_size <- 16
axis_title_size <- 18
bugs_order_var_C_summary <- bugs_order_var_C %>%
 group_by(treatment, order) %>%
 summarize(frequency = n(), .groups = "drop") %>%
 filter(frequency > 0)
ggplot(bugs_order_var_C_summary, aes(x = order, y = frequency, fill = as.factor(treatment))) +
 geom_bar(stat = "identity", position = "dodge") +
 scale_fill_manual(values = treatment_colors, name = "Treatment (gph)") +
 scale_y_log10() +
 theme_minimal() +
 theme(
  axis.text.x = element_text(angle = 45, hjust = 1, size = axis_text_size),
  axis.text.y = element_text(size = axis_text_size),
  axis.title.x = element_text(size = axis_title_size),
  axis.title.y = element_text(size = axis_title_size)
 ) +
```

```
  labs(
    x = "Order", y = "Log(Frequency)",
    title = "Log Frequency of Orders by Treatment (Variety C, 2020)"
  )
bugs_order_var_L_summary <- bugs_order_var_L %>%
  group_by(treatment, order) %>%
  summarize(frequency = n(), .groups = "drop") %>%
  filter(frequency > 0)
ggplot(bugs_order_var_L_summary, aes(x = order, y = frequency, fill = as.factor(treatment))) +
  geom_bar(stat = "identity", position = "dodge") +
  scale_fill_manual(values = treatment_colors, name = "Treatment (gph)") +
  scale_y_log10() +
  theme_minimal() +
  theme(
    axis.text.x = element_text(angle = 45, hjust = 1, size = axis_text_size),
    axis.text.y = element_text(size = axis_text_size),
    axis.title.x = element_text(size = axis_title_size),
    axis.title.y = element_text(size = axis_title_size)
  ) +
  labs(
    x = "Order", y = "Log(Frequency)",
    title = "Log Frequency of Orders by Treatment (Variety L, 2020)"
  )
bugs_family_var_C_summary <- bugs_family_var_C %>%
  group_by(treatment, family) %>%
  summarize(frequency = n(), .groups = "drop") %>%
  filter(frequency > 0)
ggplot(bugs_family_var_C_summary, aes(x = family, y = frequency, fill = as.factor(treatment))) +
  geom_bar(stat = "identity", position = "dodge") +
  scale_fill_manual(values = treatment_colors, name = "Treatment (gph)") +
  scale_y_log10() +
  theme_minimal() +
  theme(
    axis.text.x = element_text(angle = 45, hjust = 1, size = axis_text_size),
    axis.text.y = element_text(size = axis_text_size),
    axis.title.x = element_text(size = axis_title_size),
    axis.title.y = element_text(size = axis_title_size)
  ) +
  labs(
    x = "Family", y = "Log(Frequency)",
```

```
    title = "Log Frequency of Families by Treatment (Variety C, 2020)"
  )

bugs_family_var_L_summary <- bugs_family_var_L %>%
  group_by(treatment, family) %>%
  summarize(frequency = n(), .groups = "drop") %>%
  filter(frequency > 0)
ggplot(bugs_family_var_L_summary, aes(x = family, y = frequency, fill = as.factor(treatment))) +
  geom_bar(stat = "identity", position = "dodge") +
  scale_fill_manual(values = treatment_colors, name = "Treatment (gph)") +
  scale_y_log10() +
  theme_minimal() +
  theme(
    axis.text.x = element_text(angle = 45, hjust = 1, size = axis_text_size),
    axis.text.y = element_text(size = axis_text_size),
    axis.title.x = element_text(size = axis_title_size),
    axis.title.y = element_text(size = axis_title_size)
  ) +
  labs(
    x = "Family", y = "Log(Frequency)",
    title = "Log Frequency of Families by Treatment (Variety L, 2020)"
  )

################################################################################

##########ONLY USING THE LAST SAMPLING DATE FOR PLANT SIZE VARIABLES (AND NOT USING OTHER FIELD VARIABLES)
fdat2019 <- read.csv("2019_hemp_drought_field_data.csv")
fdat2020 <- read.csv("2020_drought_combined_field_data.csv")
fdat2019 <- fdat2019 %>%
  rename(treatment = water_treatment)
fdat2020 <- fdat2020 %>%
  rename(treatment = water_treatment)
fdat2019 <- fdat2019 %>%
  mutate(date = as.Date(date, format = "%m/%d/%Y"))
fdat2019 <- fdat2019 %>%
  mutate(date = gsub("^0019", "2019", date))

######2019 filter and fix date error
fdat2019 <- fdat2019 %>%
  mutate(date = as.Date(date, format = "%Y-%m-%d"))
```

```
fdat2019_last <- fdat2019 %>%
  filter(date == as.Date("2019-10-09")) %>%
  select(year, treatment, variety, replicate, Height.meters, total.leaf.count) %>%
  mutate(
    Height.meters = as.numeric(gsub("[^0-9.-]", "", Height.meters)),  # Remove non-numeric characters
    total.leaf.count = as.numeric(gsub("[^0-9.-]", "", total.leaf.count)),  # Remove non-numeric characters
    Height.meters = replace(Height.meters, is.na(Height.meters), 0),  # Replace NAs with 0
    total.leaf.count = replace(total.leaf.count, is.na(total.leaf.count), 0)  # Replace NAs with 0
  )

###### 2020 filter
fdat2020_last <- fdat2020 %>%
  filter(date == "10/6/2020") %>%
  select(year, treatment, variety, replicate, Height.meters, total.leaf.count, wind.stress) %>%
  mutate(
    Height.meters = as.numeric(gsub("[^0-9.-]", "", Height.meters)),  # Remove non-numeric characters
    total.leaf.count = as.numeric(gsub("[^0-9.-]", "", total.leaf.count)),  # Remove non-numeric characters
    wind.stress = as.numeric(gsub("[^0-9.-]", "", wind.stress)),  # Remove non-numeric characters from wind.stress
    Height.meters = replace(Height.meters, is.na(Height.meters), 0),  # Replace NAs with 0 for Height.meters
    total.leaf.count = replace(total.leaf.count, is.na(total.leaf.count), 0),  # Replace NAs with 0 for total.leaf.count
    wind.stress = replace(wind.stress, is.na(wind.stress), 0)  # Replace NAs with 0 for wind.stress
  )

field_data_combined <- bind_rows(fdat2019_last, fdat2020_last)
merged_data <- ChemDiv %>%
  merge(field_data_combined, by = c("year", "variety", "treatment", "replicate"), all.x = TRUE)

# Replace NA in "Height.meters" and "total.leaf.count" with the overall mean of each variable
merged_data <- merged_data %>%
  mutate(
    Height.meters = ifelse(is.na(Height.meters), mean(Height.meters, na.rm = TRUE), Height.meters),
    total.leaf.count = ifelse(is.na(total.leaf.count), mean(total.leaf.count, na.rm = TRUE), total.leaf.count),

    # Replace NA in "famabun" and "p" with 0
    famabun = replace(famabun, is.na(famabun), 0),
    p = replace(p, is.na(p), 0)
  )

merged_data <- merged_data %>%
  group_by(treatment, variety, replicate) %>%
```

```
  mutate(
    mean_wind_stress = mean(wind.stress, na.rm = TRUE),
    wind.stress = ifelse(is.na(wind.stress),
                         ifelse(is.na(mean_wind_stress), 0, mean_wind_stress),
                         wind.stress)
  ) %>%
  ungroup() %>%
  select(-mean_wind_stress)

########################################

ChemDivOrig <- ChemDiv
ChemDiv <- merged_data

######################Back to the full data
# I created dummy variables for variety, with 'T' as the reference
ChemDiv <- ChemDiv %>%
     mutate(variety_L = ifelse(variety == "L", 1, 0),
            variety_C = ifelse(variety == "C", 1, 0))
# I standardized only the numeric variables
ChemDiv <- ChemDiv %>%
  mutate(across(where(is.numeric) & !c(year, treatment, variety_C, variety_L, replicate, identifier), scale))

##Diversity, q=2 (simpson's) for chemistry for EACH SAMPLE
ChemDiv_compounds <- ChemDiv %>%
  select(CBGA, Delta9.THCA, CBDA, CBDV, CBD, CBN, Delta8.THC)
ChemDiv$canndiv <- apply(ChemDiv_compounds, 1, function(row) {
  row_proportions <- row / sum(row)
  1 / sum(row_proportions^2)
})

ChemDiv <- ChemDiv %>%
  mutate(
    drought = ifelse(treatment == 0.5, 1, 0),  # Drought is 1 when treatment is 0.5, else it's 0
    flood = ifelse(treatment == 2, 1, 0)       # Flood is 1 when treatment is 2, else it's 0
  )

ChemDiv_2019 <- ChemDiv %>% filter(year == 2019)
ChemDiv_2020 <- ChemDiv %>% filter(year == 2020)
```

```
##############################################SEMs with factors
###Factor analysis for 2020 to create chemistry latent variable
ChemDiv_2020_chemistry <- ChemDiv_2020 %>% select(CBDV, CBD, CBN, Delta8.THC)
fa_2020 <- fa(ChemDiv_2020_chemistry, nfactors = 1, rotate = "none")
ChemDiv_2020$Factor1_2020 <- fa_2020$scores[, 1]

model_2020 <- '
  Height.meters ~ variety_C + drought + flood + wind.stress
  richness ~ Factor1_2020 + Height.meters + variety_C + drought + flood
  Factor1_2020 ~ variety_C + drought + flood + Height.meters + wind.stress
'
fit_2020 <- sem(model_2020, data = ChemDiv_2020)
summary(fit_2020, standardized = TRUE)

# Plot for 2020
lavaanPlot(model = fit_2020,
           node_options = list(shape = "box", fontname = "Helvetica"),
           edge_options = list(color = "darkgreen"),
           coefs = TRUE,
           covs = FALSE,
           stand = TRUE)

###############################SAME SEMs USING phytochemical diversity -

model_2020 <- '
  Height.meters ~ variety_C + drought + flood + wind.stress  # Only variety C (L is the reference)
  richness ~ canndiv + Height.meters + variety_C + flood
  canndiv ~ variety_C + drought + flood + Height.meters + wind.stress
'
fit_2020 <- sem(model_2020, data = ChemDiv_2020)
summary(fit_2020, standardized = TRUE)

# Plot for 2020
lavaanPlot(model = fit_2020,
           node_options = list(shape = "box", fontname = "Helvetica"),
           edge_options = list(color = "darkgreen"),
           coefs = TRUE,
           covs = FALSE,
           stand = TRUE)
```

```
####################### parsimonious SEMs FOR CBD
model_2020 <- '
  Height.meters ~ flood + wind.stress
  richness ~ CBD + Height.meters + variety_C + flood
  CBD ~ variety_C + Height.meters + wind.stress
'
fit_2020 <- sem(model_2020, data = ChemDiv_2020)
summary(fit_2020, standardized = TRUE)

# Plot for 2020
lavaanPlot(model = fit_2020,
           node_options = list(shape = "box", fontname = "Helvetica"),
           edge_options = list(color = "darkgreen"),
           coefs = TRUE,
           covs = FALSE,
           stand = TRUE)


##########################################################################################################
##############################################################################################################
##traditional SEM   DO NOT INCLUDE
# SEM model for 2020 with varieties C and L
model_2020 <- '
  chemical_defense =~ CBGA + Delta9.THCA + CBDA + CBDV + CBD + CBN + Delta8.THC
  abundance ~ chemical_defense + variety_C + treatment  # Only variety C (L is the reference)
  richness ~ chemical_defense + abundance + variety_C + treatment
  chemical_defense ~ variety_C + treatment
'
fit_2020 <- sem(model_2020, data = ChemDiv_2020)
summary(fit_2020, standardized = TRUE)

# Plot for 2020
lavaanPlot(model = fit_2020,
           node_options = list(shape = "box", fontname = "Helvetica"),
           edge_options = list(color = "darkgreen"),
           coefs = TRUE,
           covs = FALSE,
           stand = TRUE)
```

```
###########################################################################################################
############################################################
# Metabolomics, non-targeted analyses
metabolomics <- read.csv("EKHempDrought_ISD_Decon_DWT2.csv")
metabolomics[is.na(metabolomics)] <- 0
metabolomics <- metabolomics %>%
  mutate(treatment = str_extract(identifier, "(?<=trt_)(\\d+\\.\\d+|\\d+)") %>%
         str_replace("^05$", "0.5") %>%
         as.numeric()) %>%
  mutate(variety = str_extract(identifier, "(?<=var_)\\w+")) %>%
  mutate(replicate = as.numeric(str_extract(identifier, "(?<=rep_)\\d+"))) %>%
  mutate(year = as.numeric(str_extract(identifier, "(?<=Drought\\.)\\d+"))) %>%
 mutate(part = ifelse(year == 2020, "flower", str_extract(identifier, "flower|leaf")))
metabolomics_2019 <- metabolomics %>% filter(year == 2019)
metabolomics_2020 <- metabolomics %>% filter(year == 2020)

# excluding identifier, treatment, variety, replicate, year, part
numeric_columns <- metabolomics %>%
  select(-identifier, -treatment, -variety, -replicate, -year, -part)
hill_results <- t(apply(numeric_columns, 1, calculate_hill_numbers_row))
metabolomics <- bind_cols(metabolomics, as.data.frame(hill_results))

# Group by year, part, variety, and treatment, and calculate mean and variance for Hill numbers
summary_stats <- metabolomics %>%
  group_by(year, part, variety, treatment) %>%
  summarise(
    mean_D0 = mean(D0, na.rm = TRUE),
    var_D0 = var(D0, na.rm = TRUE),
    mean_D1 = mean(D1, na.rm = TRUE),
    var_D1 = var(D1, na.rm = TRUE),
    mean_D2 = mean(D2, na.rm = TRUE),
    var_D2 = var(D2, na.rm = TRUE)
  ) %>%
  as.data.frame()

#################################################################SEM
# Select relevant columns and rename D0, D1, D2 to Met0, Met1, Met2
metabolomics_selected <- metabolomics %>%
  select(year, treatment, variety, replicate, part, D0, D1, D2) %>%
  rename(Met0 = D0, Met1 = D1, Met2 = D2)
```

```
merged_data <- ChemDiv %>%
  merge(metabolomics_selected, by = c("year", "treatment", "variety", "replicate"), all.x = TRUE)
#write.csv(merged_data, "MergedCannabis.csv", row.names = FALSE)

merged_data_2020 <- merged_data %>%
  filter(year == 2020)
merged_data_2019 <- merged_data %>%
  filter(year == 2019)

################################SAME SEMs USING UNTARGETED phytochemical diversity
##CORRELATIONS
selected_vars <- c("drought", "flood", "wind.stress", "CBD", "CBDA", "CBDV", "CBGA", "CBN", "Delta8.THC", "Delta9.THCA", "abundance", "richness",
              "famabun", "D2", "Height.meters", "total.leaf.count", "canndiv",
                "Met0", "Met1", "Met2")
numeric_data_2020 <- merged_data_2020 %>%
  select(all_of(selected_vars)) %>%
  select(where(is.numeric))
numeric_data_2019 <- merged_data_2019 %>%
  select(all_of(selected_vars)) %>%
  select(where(is.numeric))
cor_matrix_2020 <- cor(numeric_data_2020, use = "complete.obs")
ggcorrplot(cor_matrix_2020,
           method = "circle",
           type = "lower",
           lab = TRUE,
           title = "Correlation Matrix - 2020",
           lab_size = 3)


###########SEM
model_2020 <- '
  Height.meters ~ flood + wind.stress  # Only variety C (L is the reference)
  richness ~ Met1 + Height.meters + flood
  Met1 ~ variety_C + drought + wind.stress
'
fit_2020 <- sem(model_2020, data = merged_data_2020)
summary(fit_2020, standardized = TRUE)

# Plot for 2020
lavaanPlot(model = fit_2020,
```

```
        node_options = list(shape = "box", fontname = "Helvetica"),
        edge_options = list(color = "darkgreen"),
        coefs = TRUE,
        covs = FALSE,
        stand = TRUE)


##########################################################################
#######frequentist models - SEPARATED  BY YEAR
model_CBD_2020 <- lm(CBD ~ drought + flood + variety + wind.stress, data = merged_data_2020)
summary(model_CBD_2020)
model_Met1_2020 <- lm(Met1 ~ drought + flood + variety + wind.stress, data = merged_data_2020)
summary(model_Met1_2020)
model_FACTOR_2020 <- lm(Factor1_2020 ~ drought + flood + variety + wind.stress, data = ChemDiv_2020)
summary(model_FACTOR_2020)

###FOCAL MODELS
# Linear model with interactions between drought, flood, variety, and wind.stress
model_Met1_2020 <- lm(Met1 ~ drought  + flood + variety*drought + variety*flood + wind.stress, data = merged_data_2020)
summary(model_Met1_2020)

#####################bayesian summaries - CHANGE RESPONSE VARIABLE BASED ON WHAT YOU'RE ANALYZING
library(rjags)
library(coda)
library(MCMCvis)
library(bayesplot)
library(reshape2)
merged_data_2020$CBD <- as.numeric(merged_data_2020$CBD)
merged_data_2020$Met1 <- scale(merged_data_2020$Met1)
merged_data_2020$Met1 <- as.numeric(merged_data_2020$Met1)
response_variable_2020 <- merged_data_2020$Met1
#response_variable_2020 <- merged_data_2020$CBD

# Prepare data for JAGS
dataForJags_2020 <- list(
  N = length(response_variable_2020),
  response = response_variable_2020,
  drought = as.numeric(merged_data_2020$drought),
  flood = as.numeric(merged_data_2020$flood),
  wind_stress = as.numeric(merged_data_2020$wind.stress),
```

```
  variety = as.numeric(as.factor(merged_data_2020$variety))
)

# Modified model string to estimate posterior means and variances
model_string_2020 <- "
  model {
    for (i in 1:N) {
      response[i] ~ dnorm(mu[i], tau)
      mu[i] <- beta0 + beta1 * drought[i] + beta2 * flood[i] + beta3 * wind_stress[i] +
              beta4 * variety[i] + beta5 * drought[i] * variety[i] + beta6 * flood[i] * variety[i]
    }

    # Priors
    tau ~ dgamma(0.01, 0.01)  # Precision for normal distribution
    sigma <- 1 / sqrt(tau)    # Standard deviation of the response variable

    beta0 ~ dnorm(0, 0.01)
    beta1 ~ dnorm(0, 0.01)
    beta2 ~ dnorm(0, 0.01)
    beta3 ~ dnorm(0, 0.01)
    beta4 ~ dnorm(0, 0.01)
    beta5 ~ dnorm(0, 0.01)
    beta6 ~ dnorm(0, 0.01)

    # Posterior predictions for specific treatment level combinations
    mu_pred[1] <- beta0 + beta1 * 0 + beta2 * 0 + beta3 * 0 + beta4 * 2 + beta5 * 0 * 2 + beta6 * 0 * 2  # A
    mu_pred[2] <- beta0 + beta1 * 0 + beta2 * 0 + beta3 * 2 + beta4 * 2 + beta5 * 0 * 2 + beta6 * 0 * 2  # B
    mu_pred[3] <- beta0 + beta1 * 1 + beta2 * 0 + beta3 * 0 + beta4 * 2 + beta5 * 1 * 2 + beta6 * 0 * 2  # C
    mu_pred[4] <- beta0 + beta1 * 1 + beta2 * 0 + beta3 * 2 + beta4 * 2 + beta5 * 1 * 2 + beta6 * 0 * 2  # D
    mu_pred[5] <- beta0 + beta1 * 0 + beta2 * 1 + beta3 * 0 + beta4 * 2 + beta5 * 0 * 2 + beta6 * 1 * 2  # E
    mu_pred[6] <- beta0 + beta1 * 0 + beta2 * 1 + beta3 * 2 + beta4 * 2 + beta5 * 0 * 2 + beta6 * 1 * 2  # F

    mu_pred[7] <- beta0 + beta1 * 0 + beta2 * 0 + beta3 * 0 + beta4 * 1 + beta5 * 0 * 1 + beta6 * 0 * 1  # I
    mu_pred[8] <- beta0 + beta1 * 0 + beta2 * 0 + beta3 * 2 + beta4 * 1 + beta5 * 0 * 1 + beta6 * 0 * 1  # J
    mu_pred[9] <- beta0 + beta1 * 1 + beta2 * 0 + beta3 * 0 + beta4 * 1 + beta5 * 1 * 1 + beta6 * 0 * 1  # K
    mu_pred[10] <- beta0 + beta1 * 1 + beta2 * 0 + beta3 * 2 + beta4 * 1 + beta5 * 1 * 1 + beta6 * 0 * 1 # L
    mu_pred[11] <- beta0 + beta1 * 0 + beta2 * 1 + beta3 * 0 + beta4 * 1 + beta5 * 0 * 1 + beta6 * 1 * 1 # M
    mu_pred[12] <- beta0 + beta1 * 0 + beta2 * 1 + beta3 * 2 + beta4 * 1 + beta5 * 0 * 1 + beta6 * 1 * 1 # N
  }
"
```

```
# Parameters to monitor
params_2020 <- c("mu_pred", "sigma")

# Initial values for chains
init_vals_2020 <- list(
  list(beta0 = 0, beta1 = 0, beta2 = 0, beta3 = 0, beta4 = 0, beta5 = 0, beta6 = 0, tau = 1),
  list(beta0 = 0, beta1 = 0, beta2 = 0, beta3 = 0, beta4 = 0, beta5 = 0, beta6 = 0, tau = 1)
)

# Run JAGS model
n_iter <- 10000
n_burnin <- 5000
bayesian_model_2020 <- jags.model(textConnection(model_string_2020), data = dataForJags_2020, inits = init_vals_2020, n.chains = 2)
update(bayesian_model_2020, n_burnin)  # Burn-in phase
samples_2020 <- coda.samples(bayesian_model_2020, variable.names = params_2020, n.iter = n_iter)
summary(samples_2020)

posterior_means_2020 <- as.data.frame(as.matrix(samples_2020))
posterior_means_2020 <- posterior_means_2020[, grepl("mu_pred", colnames(posterior_means_2020))]
posterior_subsample <- posterior_means_2020[sample(1:nrow(posterior_means_2020), 1000), ]

combination_labels <- c("A", "B", "C", "D", "E", "F", "I", "J", "K", "L", "M", "N")
colnames(posterior_subsample) <- combination_labels
posterior_melted_2020 <- reshape2::melt(posterior_subsample)
custom_colors <- c("A" = "#CC8899",  # Muted Pink for A and I
                   "B" = "#5F9EA0",  # Muted Blue for B and J
                   "C" = "#66CDAA",  # Muted Green for C and K
                   "D" = "#DAA520",  # Darker Gold for D and L
                   "E" = "#C71585",  # Darker Hot Pink for E and M
                   "F" = "#8B3A3A",  # Darker Red for F and N
                   "I" = "#CC8899",  # Muted Pink for A and I
                   "J" = "#5F9EA0",  # Muted Blue for B and J
                   "K" = "#66CDAA",  # Muted Green for C and K
                   "L" = "#DAA520",  # Darker Gold for D and L
                   "M" = "#C71585",  # Darker Hot Pink for E and M
                   "N" = "#8B3A3A")  # Darker Red for F and N

ggplot(posterior_melted_2020, aes(x = variable, y = value, fill = variable)) +
  geom_boxplot(outlier.shape = NA, color = "black") +
```

```
  geom_jitter(width = 0.2, alpha = 0.3, aes(color = variable)) +  # Adjusted alpha for lighter points
  scale_fill_manual(values = custom_colors) +
  scale_color_manual(values = custom_colors) +  # Use the same colors for points as the boxes
  theme_minimal() +
  theme(
    axis.text.x = element_text(angle = 0, hjust = 0.5, size = 14, face = "bold"),
    axis.text.y = element_text(size = 28, face = "bold"),  # Doubled the size of y-axis numbering
    axis.title = element_text(size = 14, face = "bold"),
    panel.grid = element_blank()  # Remove gridlines
  ) +
  labs(x = "Treatment Combinations", y = "Posterior Mean Estimates",
       title = "Posterior Estimates of Response Variable under Treatment Combinations")
cat("\nLegend:\n")
legend_labels <- c(
  "A: Drought=Control, Flood=Control, Wind=Control, Variety=L",
  "B: Drought=Control, Flood=Control, Wind=High Stressed, Variety=L",
  "C: Drought=Water Deficit, Flood=Control, Wind=Control, Variety=L",
  "D: Drought=Water Deficit, Flood=Control, Wind=High Stressed, Variety=L",
  "E: Drought=Control, Flood=Excess, Wind=Control, Variety=L",
  "F: Drought=Control, Flood=Excess, Wind=High Stressed, Variety=L",
  "I: Drought=Control, Flood=Control, Wind=Control, Variety=C",
  "J: Drought=Control, Flood=Control, Wind=High Stressed, Variety=C",
  "K: Drought=Water Deficit, Flood=Control, Wind=Control, Variety=C",
  "L: Drought=Water Deficit, Flood=Control, Wind=High Stressed, Variety=C",
  "M: Drought=Control, Flood=Excess, Wind=Control, Variety=C",
  "N: Drought=Control, Flood=Excess, Wind=High Stressed, Variety=C"
)
cat(paste(legend_labels, collapse = "\n"))



###############MODEL with better variable names MET1
# Rename columns in the data to match the new variable names in the model
merged_data_2020 <- merged_data_2020 %>%
  dplyr::rename(
    water_stress = drought,
    wind_stress = wind.stress,
    arth_div = richness,
    phy_div = Met1,
    varieties = variety_C,  # Updated to "varieties"
```

```
    Height = Height.meters
  )
model_2020 <- '
  Height ~ flood + wind_stress  # Only variety C (L is the reference)
  arth_div ~ phy_div + Height + flood
  phy_div ~ varieties + water_stress + wind_stress
'
fit_2020 <- sem(model_2020, data = merged_data_2020)
summary(fit_2020, standardized = TRUE)
lavaanPlot(model = fit_2020,
           node_options = list(shape = "box", fontname = "Helvetica"),
           edge_options = list(color = "darkgreen"),
           coefs = TRUE,
           covs = FALSE,
           stand = TRUE)

####################### CBD
ChemDiv_2020 <- ChemDiv_2020 %>%
  dplyr::rename(
    water_stress = drought,
    wind_stress = wind.stress,
    arth_div = richness,
    varieties = variety_C,  # Updated to "varieties"
    Height = Height.meters
  )
model_2020 <- '
  Height ~ flood + wind_stress
  arth_div ~ CBD + Height + varieties + flood
  CBD ~ varieties + Height + wind_stress
'
fit_2020 <- sem(model_2020, data = ChemDiv_2020)
summary(fit_2020, standardized = TRUE)
lavaanPlot(model = fit_2020,
           node_options = list(shape = "box", fontname = "Helvetica"),
           edge_options = list(color = "darkgreen"),
           coefs = TRUE,
           covs = FALSE,
           stand = TRUE)
```

```
##############SORTING FOR CASEY
sorted_by_met1 <- merged_data_2020[order(-merged_data_2020$phy_div), ]
sorted_by_met2 <- merged_data_2020[order(-merged_data_2020$Met2), ]
head(sorted_by_met1)
head(sorted_by_met2)
tail(sorted_by_met1)
tail(sorted_by_met2)
```
